# Early community-based family practice elective positively influences medical students’ career considerations – a Pre-post-comparison

**DOI:** 10.1186/1471-2296-14-24

**Published:** 2013-02-21

**Authors:** Tobias Deutsch, Petra Hönigschmid, Thomas Frese, Hagen Sandholzer

**Affiliations:** 1 Department of Primary Care, Leipzig Medical School, Philipp-Rosenthal-Str. 55, Leipzig 04103, Germany

**Keywords:** Career choice, Family practice, Elective, Medical education

## Abstract

**Background:**

Demographic change and recruitment problems in family practice are increasingly threatening an adequate primary care workforce in many countries. Thus, it is important to attract young physicians to the field. The purpose of the present study was to examine the effect of an early community-based 28-h family practice elective with one-to-one mentoring on medical students’ consideration of family practice as a career option, their interest in working office-based, and several perceptions with regard to specific aspects of a family physician’s work.

**Methods:**

First- and second-year medical students completed questionnaires before and after a short community-based family practice elective, consisting of a preparatory course and a community-based practical experience with one-to-one mentoring by trained family physicians.

**Results:**

We found a significantly higher rate of students favoring family practice as a career option after the elective (32.7% vs. 26.0%, p = 0.039). Furthermore, the ranking of family practice among other considered career options improved (p = 0.002). Considerations to work office-based in the future did not change significantly. Perceptions regarding a family physician’s job changed positively with regard to the possibility of long-term doctor-patient relationships and treatment of complex disease patterns. The majority of the students described identification with the respective family physician tutor as a professional role model and an increased interest in the specialty.

**Conclusions:**

Our results indicate that a short community-based family practice elective early in medical education may positively influence medical students’ considerations of a career in family practice. Furthermore, perceptions regarding the specialty with significant impact on its attractiveness may be positively adjusted. Further research is needed to evaluate the influence of different components of a family practice curriculum on the de facto career decisions of young physicians after graduation.

## Background

As in many other Western industrial countries, there is an increasing shortage of family physicians in Germany [1-3]. The retirement age of office-based family physicians is rising and there is a growing proportion of office-based family physicians aged 60 years and over. There is a need for action [1]. Furthermore, the number of young physicians who completed a residency in family practice has decreased over the last years [4]. Thus, it is important to attract young physicians to family practice and to increase the rate of those who decide to work office-based. In addition to modifications of the respective framework on a political level, the influence of medical schools might play a crucial role. The investigation of medical students’ career considerations, its determinants, and the evaluation of possibilities to exert influence consequently constitutes a growing field of interest and research. It is known from previous studies that demographic characteristics like gender, age, relationship status, parental socio-economic status, family or friends in medicine and regional background are associated with the choice of a career in family practice [5-9]. Moreover, the career aspiration at study entry [5,8] and personal priorities regarding interest in research, income, prestige, work-life-balance, a varied scope of practice and patients, and long-term doctor-patient relationships exert a significant impact on the career choice [5,8,10-12].

While, on the one hand, approaches to increase medical schools’ output of students choosing family practice/primary care careers via selection or targeting of medical students based on characteristics demonstrably associated with career considerations or career choice are discussed, other approaches aim to convince medical students to become family physicians [6,13].

In this regard, the investigation of the respective effect of specific curricular components is of exceptional interest. Practical experiences in family practice during medical education, mandatory family practice clerkships (desirably over several weeks), rural practice experiences, longitudinal primary care tracks or mentoring, and positive role-models seem to have the potential to attract young medical students to family practice [5,6,9,14-17]. In addition, the influence of the “hidden curriculum”, the “corporate culture” of a medical school regarding the specialty of family practice, should not be underestimated [5,6,10]. As the number of students considering a career in family practice seems to drop especially within the first years in medical school [18], some authors emphasize the potential benefit of interventions early in medical education [14,18].

The present study examined the effect of an early community-based 28-h family practice elective with one-to-one mentoring on medical students’ consideration of family practice as a career option and their interest in working office-based. In addition, the effect on several perceptions with regard to aspects of a family physician’s work was of interest, since misconceptions about the specialty influence students’ career considerations [19], but might be changed by undergraduate exposures to family practice [20,21].

## Methods

### Sampling and design

The present study was set up as a pre-post-study. Data were collected between February 2008 and February 2010. In total, 140 medical students from Leipzig Medical School in their first and second year (of six) who took part in a preclinical family practice elective completed questionnaires before and after. The preclinical family practice elective has been offered at Leipzig Medical School for the last ten years to attract students to the specialty of family practice early in medical education. It covers 28 h, consisting of a preparatory course in the form of a seminar (7 h) and a community-based experience with one-to-one mentoring by trained family physicians collaborating with Leipzig Medical School (21 h, 3 workdays). Within the preparatory course, students are instructed in taking medical history and performing simple physical examinations like measuring blood pressure and otoscopy, and have the opportunity to train these skills. During the community-based experience, students assist the family physician, take medical histories and apply their new examination skills. Furthermore, a medical report regarding a home-visit patient has to be written.

### Questionnaire

The questionnaire used in the present study was self-constructed. Before and after the elective, students were asked for information about their considerations concerning the following aspects: current career aspiration (main), current career considerations (up to four considerations, ranked), future clerkship, one-third of practical year (the practical year is the final year of the German medical education and is divided in three thirds: surgery (mandatory), internal medicine (mandatory) and one other specialty selected by the student), or dissertation in family practice, and working office-based in the future. Furthermore, they were asked to assess statements with regard to aspects of a family physician’s work on four-point Likert-scales (1 = “do not agree at all”, 2 = “rather disagree”, 3 = “rather agree”, 4 = “strongly agree”; and a fifth category “don’t know”). The statements to be assessed were based on previous studies on the consideration of a career in family practice and refer to working time, workload, work-life-balance, varied scope of practice, opportunity to have long-term doctor-patient relationships and to treat complex disease patterns [11,12,16,19,22-24]. Additionally, students were asked to evaluate the elective after completion (using again the mentioned four-point Likert-scales) with regard to the following aspects: identification with the family physician’s working style, finding a role model, awakened interest concerning family practice, and increased motivation to become a family physician. To characterize the student sample, relevant demographic information like age, gender, current preclinical study semester, regional background, previous experiences in family practice, medical educational background, and family or friends in family practice were collected prior to the elective.

### Statistical analysis

The anonymized data analysis was performed with IBM® SPSS® Statistics 18.0 Software for Windows. For the pre-post-comparisons, we used McNemar’s test for dichotomous nominal variables, Marginal Homogeneity test to evaluate change in multinomial data, and Wilcoxon signed rank test to evaluate differences in central tendency. The number of persons analyzed within the different pre-post comparisons varied due to missing values or, in some cases, due to the extent of the use of the category “don’t know”. Statistical significance was assumed at a probability of error of p < 0.05.

### Ethical approval

Our investigation was in accordance to the declaration of Helsinki. According to the regulations of the ethics committee of the Leipzig Medical School an explicit ethical approval was deemed not necessary.

## Results

From 140 students who were originally enrolled for the elective, three dropped out due to illness and four for unknown reasons. The remaining 133 persons could be analyzed. The sample characteristics are presented in Table 1.

**Table 1:** Sample characteristics

**Characteristic**	**N**	Percent (n) *
female sex	133	75.9 (101)
age *(yr, mean (SD), min - max)*	133	21.4 (3.5), 18–47
semester	133	
1^st^		64.7 (86)
3^rd^		34.6 (46)
4^th^		0.8 (1)
regional background	132	
major city		32.6 (43)
provincial town		38.6 (51)
countryside		28.8 (38)
previous experiences in family practice	133	12.8 (17)
medical educational background	133	18.0 (24)
family or friends in family practice	133	25.6 (34)

* unless otherwise indicated.

We found no relevant or statistically significant pre-post difference in the students’ consideration to work office-based in the future (47.4 vs. 51.9%, p = 0.286, n = 133) or to complete a further clerkship (92.4 vs. 90.9%, p = 0.774, n = 132), a third of their practical year (85.4 vs. 82.3%, p = 0.454, n = 130) or a dissertation in family practice (70.4 vs. 70.4%, p = 1.000, n = 125).

A significant pre-post-difference could be found in the number of students declaring family practice to be their favored career option (26.0 vs. 32.7%, p = 0.039, n = 104). Table 2 provides a more detailed view on the students’ considerations of family practice as a career option: the ranking of family practice as a career option was significantly better after the elective. The number of students who did not consider a career in family practice prior to the elective decreased remarkably from 28.8 to 18.3%.

**Table 2:** Rank of family practice as one career option among several considerations

**Rank of family practice**	**Pre (%)**	**Post (%)**	**p**
1^st^ (favored) career option	26.0	32.7	0.002
2^nd^ career option	26.9	27.9	
3^rd^ career option	12.5	17.3	
4^th^ career option	5.8	3.8	
family practice is actually no career option	28.8	18.3	

(Marginal Homogeneity test, N = 104).

The students’ perceptions regarding a family physician’s work changed significantly for the aspects “possibility of long-term doctor-patient relationships”, the “treatment of complex disease patterns” and “estimated working time per week” (see Table 3 for details).

**Table 3:** Perceptions with regard to aspects of a family physician’s work – Pre-Post-comparisons

**Statement**	**Agreement pre**	**Agreement post**	**N**	**p**	**Post increased agreement**	**Post decreased agreement**	**Agreement remained constant**
	median (mean*) **	median (mean*) **		**(Wilcoxon)**	**n (%)**	**n (%)**	**n (%)**
The weekly amount of the working time of a family physician is controllable.	2 (2.50)	3 (2.52)	119	0.803	32 (26.9%)	29 (24.4%)	58 (48.7%)
As a family physician it is feasible to organize yourself a good work-life-balance.	3 (2.77)	3 (2.78)	122	0.914	28 (23.0%)	25 (20.5%)	69 (56.6%)
Working as a family physician provides the possibility of long-term doctor-patient relationships.	4 (3.82)	4 (3.95)	132	< 0.001	19 (14.4%)	2 (1.5%)	111 (84.1%)
Working as a family physician provides the possibility to treat complex disease patterns.	3 (3.12)	3 (3.39)	113	< 0.001	40 (35.4%)	14 (12.4%)	59 (52.2%)
There is a broad scope of disease patterns a family physician is faced with in his daily work.	4 (3.50)	4 (3.60)	124	0.086	27 (21.8%)	16 (12.9%)	81 (65.3%)
What do you estimate: what is the average weekly working time of a family physician (in hours)? [mean ± SD]	49.53 ± 6.25	53.48 ± 8.64	127	< 0.001			

* presented for better illustration of the pre-post changes.

** unless otherwise indicated.

(Scale from 1 = “do not agree at all” to 4 = “strongly agree”).

The students’ evaluations concerning the elective revealed that the majority could identify with the respective family physician’s working style and perceived the family physician tutor as a professional role model. Most students felt motivated to become a family physician and declared aroused interest in the specialty due to the elective (data not shown).

## Discussion

The present study evaluated the effect of an early community-based 28-h family practice elective with one-to-one mentoring on medical students’ consideration of family practice as a career option and several perceptions with regard to specific aspects of a family physician’s work. We found a significantly higher rate of students considering family practice as a career option after the elective.

The present sample appears to be representative for German first- and second-year students with regard to age. Female students were slightly over-represented in our sample at 75.9% (compared to 63.5% at study entry in 2008/2009 in Germany) [25]. This might be explained by the participation in the elective by choice and a higher interest in family practice by female medical students [5-7,14]. In our sample, slightly more than one-quarter of the students already favored a career in family practice previous to the elective. This proportion is difficult to interpret since the data reported by others vary in a broad range depending on country, region, and medical school [18,22,26,27]. For the German context, it seems to be relatively high, probably again due to the participation in the elective by choice.

Previous study results concerning the influence of interventions especially early in medical education on medical students’ career considerations are inhomogeneous. While, for example, Senf et al. (2003) conclude from their literature review minimal or no effect [10], Corbett and colleagues found a modest impact of a second-year primary care preceptorship on students’ considerations of generalist careers with a significant relationship to career choice at graduation [28]. Bunker and Shadbolt (2009) concluded from their review that appropriately timed, relevant, positive exposures to general practice and its practitioners may lead to more individuals considering it as a career choice [9]. Also the findings of Howe and Ives (2001) support the hypothesis of respective advantages of shifting medical education to primary care settings [29]. Dornan et al. (2006) concluded from their review that exposure to community settings early in medical education helps medical students to socialize with their chosen profession and can influence career choices [30]. As supported by the cited investigations, our data suggest an impact of an early community-based family practice elective with one-to-one mentoring on medical students’ current consideration of family practice as a career option. Furthermore, the ranking of family practice among other considered career options improved. Particularly worth mentioning is that the number of students who could not imagine a career in family practice at all decreased.

In contrast, we could not find any changes concerning the consideration of family practice with regard to a future clerkship, one-third of practical year, or dissertation. This may be caused by the relatively high openness of the students towards family practice already previously to the elective. These findings are in accordance with those of Hill-Sakurai et al. (2003), who found that a required preclinical course work with family physicians did not increase the amount or quality of interested students’ interaction with family practice faculty [31]. Although slightly higher after the elective on a descriptive level, we found no statistically significant changes regarding the wish to work office-based in the future. It might be possible that this consideration is too demanding at this stage of studies. We found positive changes in the perceptions regarding the possibility of having long-term doctor-patient relationships and of treating complex disease patterns when working as a family physician. This appears to be important because, according to previous studies, respective perceptions are associated with the attractiveness of a career in family practice [8,12,19,22,23]. Furthermore, other authors described that medical students’ perceptions of family practice change during medical school, partially explainable by greater contact with family physicians within community-based curriculum [32,33]. Although the students in our sample adjusted their estimation regarding the average weekly working time of a family physician significantly upwards, their perceptions concerning the manageability of a family physician’s working time and the possibility to organize a positive work-life balance did not change. Especially the job conditions of family physicians in terms of family compatibility and work-life balance were frequently mentioned as relevant factors regarding the attractiveness of the specialty [11,12]. After the elective, most students felt motivated to become a family physician and declared aroused interest in the specialty.

### Limitations of the study

The present study has some limitations. One is that the results allow no conclusion with regard to a long-term effect of the elective. However, Corbett et al. (2002) found a significant relationship between career interest change due to an early family practice preceptorship and career choice at graduation [28]. Furthermore, it seems to be important to protect and cultivate interest in a career in family practice over the time of medical studies [18]. The participation in the elective by choice must also be discussed. This might have led to an over-representation of students generally interested in family practice within our sample, but it can be asked if this is a matter of serious concern. One might argue that an important contribution to increasing the number of students who decide for family practice after graduation is to maintain the interest of those who could initially imagine a family practice career during the course of their studies [5,18]. Moreover it must be pointed out that almost 29% of the students in our sample initially did not consider becoming a family physician at all. Another 45% considered the specialty initially, but favoured another one. This indicates that a noticeable number of students not or at least not definitely interested in the specialty nevertheless take part in respective offers by choice, maybe because of the possibility to obtain practical experience early in medical school. These students might be “convinced”. Finally, the absence of a control group in our study should be critically discussed. Nevertheless, we consider it plausible that the changes we found were caused by the intervention.

## Conclusions

Our results indicate that a short community-based family practice elective early in medical education positively influences medical students’ considerations of a career in family practice. Furthermore, perceptions regarding the specialty with significant impact on its attractiveness may be positively adjusted. Overall, the present findings strengthen the evidence that community-based exposures to family practice early in medical education may be a relevant component within a strategy to awake or maintain medical students’ interest in a career in family practice over the course of studies. Further research should extent the evidence on the current influence of different components of the family practice curriculum, voluntary and mandatory, on the de facto career decisions of young physicians after graduation.

## Competing interests

The authors declare that they have no competing interests.

## Authors’ contributions

TD contributed to conception and design, data analysis and interpretation, and drafted the manuscript. PH was local coordinator of the study, adapted the intervention to local needs, contributed to educational intervention, carried out the data acquisition, and revised the manuscript. TF contributed to data interpretation and revised the manuscript. HS conceived of the study design in general and the educational intervention in general practice, and contributed to data analysis and interpretation. All authors read and approved the final manuscript.

## Pre-publication history

The pre-publication history for this paper can be accessed here:

http://www.biomedcentral.com/1471-2296/14/24/prepub

## References

[B1] Kopetsch T Dem deutschen Gesundheitswesen gehen die Ärzte aus! Studie zur Altersstruktur- und Arztzahlentwicklung 2010 5 Berlin: Kassenärztliche Bundesvereinigung

[B2] Pugno PA McGaha AL Schmittling GT DeVilbiss Bieck AD Crosley PW Ostergaard DJ Results of the 2010 national resident matching program: family medicine Fam Med 2010 42 552 561 20830620

[B3] Vanasse A Orzanco MG Courteau J Scott S Attractiveness of family medicine for medical students: influence of research and debt Can Fam Physician 2011 57 e216 227 21673198 PMC3114693

[B4] Bundesaerztekammer: Anzahl der erteilten Anerkennungenhttp://www.bundesaerztekammer.de/page.asp?his=0.3.9237.9244

[B5] Bennett KL Phillips JP Finding, recruiting, and sustaining the future primary care physician workforce: a new theoretical model of specialty choice process Acad Med 2010 85 Suppl 81 88 10.1097/ACM.0b013e3181ed4bae 20881711

[B6] Bland CJ Meurer LN Maldonado G Determinants of primary care specialty choice: a non-statistical meta-analysis of the literature Acad Med 1995 70 620 641 10.1097/00001888-199507000-00013 7612128

[B7] Jeffe DB Whelan AJ Andriole DA Primary care specialty choices of United States medical graduates, 1997–2006 Acad Med 2010 85 947 958 10.1097/ACM.0b013e3181dbe77d 20505392

[B8] Scott I Gowans M Wright B Brenneis F Banner S Boone J Determinants of choosing a career in family medicine CMAJ 2011 183 E1 8 20974721 10.1503/cmaj.091805 PMC3017271

[B9] Bunker J Shadbolt N Choosing general practice as a career - the influences of education and training Aust Fam Physician 2009 38 341 344 19458806

[B10] Senf JH Campos-Outcalt D Kutob R Factors related to the choice of family medicine: a reassessment and literature review J Am Board Fam Pract 2003 16 502 12 10.3122/jabfm.16.6.502 14963077

[B11] Kiolbassa K Miksch A Hermann K Loh A Szecsenyi J Joos S Goetz K Becoming a general practitioner - which factors have most impact on career choice of medical students? BMC Fam Pract 2011 12 25 10.1186/1471-2296-12-25 21549017 PMC3112095

[B12] Buddeberg-Fischer B Stamm M Buddeberg C Klaghofer R Young physicians' view on factors that increase the attractiveness of general practice Gesundheitswesen 2008 70 123 128 10.1055/s-2008-1062721 18415919

[B13] Deutsch T Hönigschmid P Wippermann U Frese T Sandholzer H Don't select medical students - convince them CMAJ 2017 2011 183 10.1503/cmaj.111-2084 PMC3225428 22106108

[B14] Compton MT Frank E Elon L Carrera J Changes in U.S. medical students' specialty interests over the course of medical school J Gen Intern Med 2008 23 1095 1100 10.1007/s11606-008-0579-z 18612751 PMC2517937

[B15] Meurer LN Influence of medical school curriculum on primary care specialty choice: analysis and synthesis of the literature Acad Med 1995 70 388 397 10.1097/00001888-199505000-00015 7748384

[B16] Jordan J Brown JB Russell G Choosing family medicine. What influences medical students? Can Fam Physician 2003 49 1131 1137 14526865 PMC2214282

[B17] Barrett FA Lipsky MS Lutfiyya MN The impact of rural training experiences on medical students: a critical review Acad Med 2011 86 259 263 10.1097/ACM.0b013e3182046387 21169781

[B18] Bethune C Hansen PA Deacon D Hurley K Kirby A Godwin M Family medicine as a career option: how students' attitudes changed during medical school Can Fam Physician 2007 53 881 885 17872751 PMC1949175

[B19] Shadbolt N Bunker J Choosing general practice - a review of career choice determinants Aust Fam Physician 2009 38 53 55 19283237

[B20] Dixon AS Lam CL Lam TP Does a brief clerkship change Hong Kong medical students' ideas about general practice? Med Educ 2000 34 339 347 10.1046/j.1365-2923.2000.00554.x 10760117

[B21] Ozcakir A Yaphe J Ercan I Perceptions of family medicine and career choice among first year medical students: a cross-sectional survey in a Turkish medical school Coll Antropol 2007 31 595 600 17847945

[B22] Wright B Scott I Woloschuk W Brenneis F Bradley J Career choice of new medical students at three Canadian universities: family medicine versus specialty medicine CMAJ 2004 170 1920 1924 15210640 10.1503/cmaj.1031111 PMC421719

[B23] Sinclair HK Ritchie LD Lee AJ A future career in general practice? A longitudinal study of medical students and pre-registration house officers Eur J Gen Pract 2006 12 120 127 10.1080/13814780600780833 17002960

[B24] Natanzon I Szecsenyi J Götz K Joos S The image of general practitioners’ profession in a changing society Med Klin (Munich) 2009 104 601 607 10.1007/s00063-009-1131-6 19701730

[B25] Bundesamt S Studierende an Hochschulen – Wintersemester 2008/2009 2009 Wiesbaden: Statistisches Bundesamt

[B26] Dikici MF Yaris F Topsever P Tuncay Muge F Gurel FS Cubukcu M Gorpelioglu S Factors affecting choice of specialty among first-year medical students of four universities in different regions of Turkey Croat Med J 2008 49 415 420 10.3325/cmj.2008.3.415 18581621 PMC2443626

[B27] Sönnichsen A Donner-Banzhoff N Baum E Motive, Berufsziele und Hoffnungen von Studienanfängern im Fach Medizin ZFA 2005 81 222 225

[B28] Corbett EC Jr Owen JA Hayden GF Effect of a second-year primary care preceptorship on medical students' career plans South Med J 2002 95 691 694 12144073

[B29] Howe A Ives G Does community-based experience alter career preference? New evidence from a prospective longitudinal cohort study of undergraduate medical students Med Educ 2001 35 391 397 10.1046/j.1365-2923.2001.00866.x 11319005

[B30] Dornan T Littlewood S Margolis SA Scherpbier A Spencer J Ypinazar V How can experience in clinical and community settings contribute to early medical education? A BEME systematic review Med Teach 2006 28 3 18 10.1080/01421590500410971 16627313

[B31] Hill-Sakurai LE Schillinger E Rittenhouse DR Fahrenbach R Hudes ES LeBaron S Shore WB Hearst N Do required preclinical courses with family physicians encourage interest in family medicine? Fam Med 2003 35 579 584 12947521

[B32] Lynch DC Newton DA Grayson MS Whitley TW Influence of medical school on medical students' opinions about primary care practice Acad Med 1998 73 433 435 10.1097/00001888-199804000-00019 9580723

[B33] Henderson E Berlin A Fuller J Attitude of medical students towards general practice and general practitioners Br J Gen Pract 2002 52 359 363 12014532 PMC1314290

